# Abnormalities on Spinal Magnetic Resonance Imaging in Children and Adolescents: A Two-Center Retrospective Cohort Study

**DOI:** 10.3390/children13020294

**Published:** 2026-02-20

**Authors:** Heshen Delwar, Nina M. C. Mathijssen, Joost H. van Linge

**Affiliations:** 1 Department of Orthopedic Surgery, Reinier de Graaf Gasthuis, 2625 AD Delft, The Netherlands; j.h.vanlinge@hagaziekenhuis.nl; 2 Department of Orthopedic Surgery, HagaZiekenhuis—Juliana Kinderziekenhuis, 2545 AA The Hague, The Netherlands; 3 Department of Orthopedic Surgery, Reinier Haga Orthopedic Center, 2725 NA Zoetermeer, The Netherlands

**Keywords:** magnetic resonance imaging, back pain, prevalence, pediatric spine, spinal abnormalities, children, adolescents

## Abstract

**Highlights:**

**What are the main findings?**

- Only 19.2% of spinal MRIs in children and adolescents revealed clinically relevant abnormalities related to symptoms.
- Patients with neurological abnormalities, a shorter symptom duration, or specific clinical indications other than chronic back pain were significantly more likely to have MRI findings that provided added diagnostic value.

**What are the implications of the main findings?**

- Routine use of spinal MRI in children and adolescents for back pain or other clinical indications has limited diagnostic yield and should be carefully considered.
- Clear criteria are needed to guide MRI referral in children and adolescents to reduce unnecessary imaging and healthcare costs.

**Abstract:**

**Background/Objectives:** Magnetic Resonance Imaging (MRI) is frequently used to evaluate back pain and other spinal indications in the pediatric population. However, the diagnostic value in the pediatric population remains unclear. This study aimed to determine the prevalence of spinal abnormalities detected by MRI in children and adolescents and to identify factors associated with MRI findings of added diagnostic value. **Methods:** A retrospective two-centre cohort study was conducted among 229 patients aged 0–16 years who underwent spinal MRI at two hospitals. MRI findings were classified into five categories: (1) no finding; (2) spinal incidental finding; (3) confirmed diagnosis with no additional information; (4) confirmed diagnosis/severity with additional information; and (5) new diagnosis. In categories 4 and 5, there was an added value of the MRI scan. Patients with and without added MRI findings were compared regarding age, gender, presence of night pain, exercise-dependent pain, sharp pain localization, trauma, neurological abnormalities, and symptom duration. **Results:** The prevalence of MRI abnormalities related to the patient’s complaints was 19.2%. When the ‘added value of MRI’ group is compared to the ‘no added value of MRI group’, neurological abnormalities (*p* = 0.009) and shorter symptom duration (*p* = 0.002) were statistically associated with abnormal MRI findings. Stratified analysis showed that MRIs provided added diagnostic value more frequently in patients with clinical indications other than chronic back pain. Most abnormalities were located in the lumbar spine, with spondylolysis/spondylolisthesis and discopathy as the most common findings. **Conclusions:** Although spinal MRIs frequently detected abnormalities, only a small proportion of MRIs revealed findings that provided added diagnostic or therapeutic value. This highlights the importance of developing clear criteria for spinal MRI use in children and adolescents to minimize unnecessary imaging, limit patient burden, and optimize healthcare resources.

## 1. Introduction

In recent years, there has been increased attention to the prevalence and impact of back pain in children and adolescents [1]. Back pain affects the quality of life in the pediatric population, interfering with daily activities, school attendance, and overall well-being [2]. The prevalence of back pain in children and adolescents varies widely; a systematic review found a lifetime prevalence of 7–72% [3]. This wide variation is influenced by differences in study populations, back pain definitions, study designs, and data collection methods [3]. Furthermore, the number of pediatric patients presenting with back pain appears to be increasing, potentially influenced by factors such as rising rates of childhood obesity, increased screen time, and the consequences of the COVID-19 pandemic, such as social distancing, isolation, and reduced physical activity [4,5,6].

Determining the cause of back pain begins with a thorough medical history and physical examination [7]. Although most causes of back pain in children are benign, serious diagnoses must not be overlooked and require further evaluation. In addition to back pain, spinal MRI in children and adolescents is also performed for other clinical indications, including neurological abnormalities, trauma, infection, or suspected malignancy [8]. Radiological imaging is often the first diagnostic tool used for evaluation of the spine. These images help to identify abnormalities such as vertebral alignment, narrowed discs, and endplate changes [9]. However, radiological imaging is insufficient for distinguishing soft-tissue lesions, necessitating the use of advanced imaging techniques. Magnetic resonance imaging (MRI) is an important advanced diagnostic tool that offers detailed visualization of the vertebrae, spinal cord, and intervertebral disc. MRI can detect abnormalities such as spondylodiscitis, herniated discs, endplate changes, spondylolysis, and in rare cases, tumours [9,10]. However, MRIs are expensive, may have limited availability, and often require sedation or general anesthesia for anxious patients or young children under 5 years of age. These factors contribute to increased costs and complexity of using MRIs [11,12,13]. Accordingly, it is both relevant and justified to question the extent to which the MRI scan provides additional clinical value.

While MRI overuse is well-documented in the adult population [14,15,16], there are limited studies examining the outcomes of spinal MRIs performed on children and adolescents. Therefore, studying the utilization of spinal MRI in children and adolescents is important to determine its effectiveness across different clinical indications and to use this knowledge to reduce unnecessary imaging and healthcare costs in the future. This study aims to assess the added diagnostic value of spinal MRI in children and adolescents referred for various clinical indications by determining the prevalence of detected abnormalities and identifying factors associated with MRI findings of added diagnostic value.

## 2. Materials and Methods

### 2.1. Study Design and Participants

This retrospective two-centre cohort study was performed in Reinier de Graaf Hospital (RDGG) and Juliana Children’s Hospital (JKZ). Spinal MRIs were performed in both centres. All MRI findings were based on the original clinical radiology reports generated at the time of imaging. No centralized or retrospective re-review of the images was performed. Patients were included if they underwent a spinal MRI and consulted the pediatric orthopedic surgeon for the same medical issue, regardless of the order in which these occurred. Indications for MRI included back pain as well as other clinical indications such as neurological abnormalities, scoliosis, congenital vertebral anomalies, trauma, infection, or suspected malignancy. Children and adolescents aged 0 to 16 years at the time of the MRI scan were eligible for inclusion. Data were retrieved from patient records available in the Electronic Health Records (EHR) systems from their implementation at each hospital until the first of March 2025. EHR implementation began in March 2012 at one hospital and in June 2016 at the other hospital. All consecutive patients who met the inclusion criteria were included. Exclusion criteria were incomplete or missing documentation of the consultation or MRI report, sacral MRIs, and multiple MRIs for the same presenting complaint; only the first MRI was included in these cases.

### 2.2. Data Collection

Patients were identified using an automated search in CTcue (IQVIA Patient Finder Solution-CTcue b.v., Amsterdam, the Netherlands). Subsequently, data were obtained from EHR and collected into electronic case report forms (eCRFs) in Castor EDC [17]. To ensure patient confidentiality, all collected data were pseudonymized using a unique code for each patient. The primary study outcome was the presence or absence of MRI abnormalities. The following data were collected from EHR: age, gender, presence of night pain, exercise-dependent pain, sharp localization of pain, relation to trauma, neurological abnormalities, duration of symptoms, imaging reports, diagnosis, and follow-up policy.

**Figure 1:**
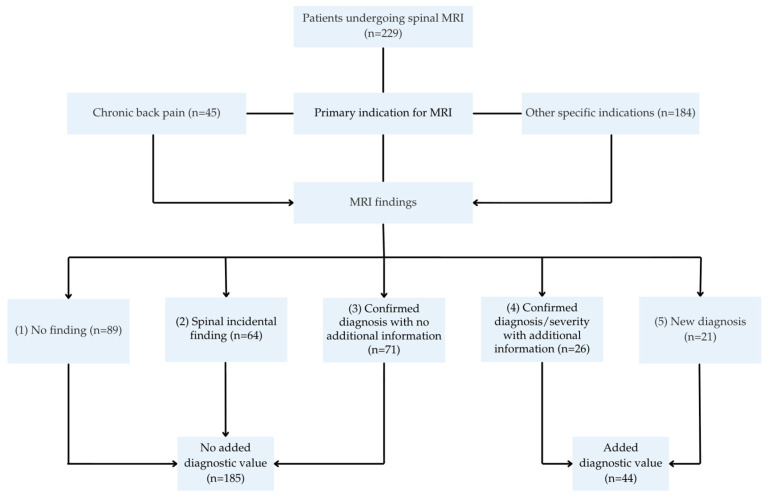
Flowchart of study workflow and classification of MRI findings by diagnostic value. Arrows indicate the flow of patients through the workflow and the classification steps. Abbreviations: MRI: Magnetic Resonance Imaging.

As back pain was frequently reported in this cohort, patients were additionally classified based on the presence or absence of back pain. For patients reporting back pain, the following variables were determined: night pain, exercise-dependent pain, sharp localization of pain, history of trauma, neurological abnormalities, and duration of symptoms. These symptom-specific variables were only applicable to patients presenting with back pain. Neurological abnormalities and a history of trauma were determined for patients without back pain. For each MRI, the presence of abnormalities was assessed based on the radiology report and categorized as “yes” or “no”. MRI abnormalities were defined as any structural or pathological finding observed on imaging. Subsequently, documentation from the radiologist and the orthopedic surgeon was reviewed to determine whether the MRI findings were related to the patient’s complaints. These findings were classified into five categories: (1) no finding; (2) spinal incidental finding; (3) confirmed diagnosis with no additional information; (4) confirmed diagnosis/severity with additional information; and (5) new diagnosis. Category 2 referred to abnormalities not related to the patient’s symptoms. Categories 3 and 4 included cases in which the abnormality had already been identified on conventional radiography. Category 3 represented cases where MRI provided no additional information, whereas category 4 represented cases where MRI was required to confirm the diagnosis or determine its severity. The added diagnostic value of MRI was defined as findings that contributed to diagnostic confirmation or establishment, or that had therapeutic consequences. Based on this definition, only MRI findings classified as categories 4 or 5 were considered to provide added diagnostic value. It is important to note that patients in the added value of MRI group may have multiple MRI abnormalities, not all of which were associated with the patient’s symptoms. Only those that were associated with the patient’s symptoms were included in the analysis. All MRI findings not classified as categories 4 or 5 were considered to have no added diagnostic value. Finally, the characteristics of patients the added-value MRI group were compared with those in the no-added-value MRI group. The study workflow is summarized in Figure 1.

### 2.3. Statistical Analyses

All statistical analyses were performed using IBM SPSS version 25.0 (IBM Corp Armonk, NY, USA). Descriptive statistics were used to summarize the demographic variables. Nominal and ordinal data were presented as counts (*n*) and percentages (%). Continuous variables that were not normally distributed were presented as medians and ranges. Group comparisons for non-normally distributed continuous variables were performed using a Mann–Whitney U-test. Categorical variables were compared using the chi-squared test. If the assumptions for the chi-squared test were not met, Fisher’s exact test or the Freeman–Halton extension of Fisher’s exact test was used. *p*-values <  0.05 were considered statistically significant.

## 3. Results

### 3.1. Baseline Demographics

A total of 229 patients met the inclusion criteria. Of these, 199 (86.9%) presented with back pain. The median age at the time of MRI was 13 years (range: 0–16 years), and 65.9% of the patients were girls. The most frequently reported symptom was exercise-dependent pain (65.3%), followed by neurological abnormalities (32.3%) and night pain (30.7%). Among patients with neurological abnormalities, radiating pain (75.7%) was the most frequently reported symptom. In 32.1% of these cases, the pain radiated below the knee. Additionally, 54.8% of patients experienced back pain for more than one year. Table 1 summarizes the descriptive statistics for all variables. The indications for requesting an MRI are presented in Table 2.

### 3.2. MRI Outcomes

A total of 229 spinal MRIs were analyzed: 89 showed no abnormalities (group 1), 64 revealed incidental findings (group 2), 71 confirmed a previously known diagnosis with no additional information (group 3), 26 confirmed the diagnosis or severity with additional information (group 4), and 21 established a new diagnosis (group 5). 44 MRIs (19.2%) showed abnormalities related to the patients’ complaints, corresponding to the added diagnostic value groups 4 and 5. Among the 44 patients, the most common abnormalities included spondylolysis/-listhesis (27.3%), discopathy (15.9%), and herniated disc (13.6%). Most abnormalities were located in the lumbar region (n = 48). The number of MRI abnormalities and findings related to the patient’s complaints are shown in Table 3 and Table 4. A comprehensive overview of all reported abnormalities and the follow-up policy of the 229 patients is provided in Supplementary Tables S1 and S2.

To explore whether the diagnostic yield varied by clinical indication, we stratified the cohort into two groups: chronic back pain (n = 45) and other specific indications (n = 184). Among these patients, only 2 MRIs in the chronic back pain group showed added diagnostic value, compared to 42 in the other indications group (*p =* 0.005). The corresponding number needed to image (NNI) was 23 for chronic back pain and 4 for other indications. Detailed results are shown in Table 5.

### 3.3. Comparison Between Groups

Table 6 presents the comparison of variables between the no-added-value MRI group and the added-value MRI group. Neurological abnormalities and pain duration were the only variables that differed significantly between the groups. Neurological abnormalities were more frequently reported in the added value of MRI group (no added value of MRI: 27.6%; added value of MRI: 52.3%, *p* = 0.009). Specifically, radiating pain (*p* = 0.015), a positive Lasègue’s sign (*p* = 0.001), and gait abnormalities (*p* = 0.050) were significantly more prevalent in the added value of MRI group. Furthermore, patients in the added value of MRI group were significantly less likely to report back pain lasting longer than one year (no added value of MRI: 58.0%; added value of MRI: 40.5%, *p* = 0.002).

## 4. Discussion

This two-centre retrospective cohort study determined the prevalence of spinal abnormalities detected by MRI in children and adolescents undergoing spinal MRI for various clinical indications. Only 19.2% of MRIs showed added diagnostic value. In comparison, Ramirez et al. reported that 50% of MRIs showed relevant findings [18], and Feldman et al. reported 53% [19]. However, another study found that 64% of MRIs showed abnormalities; only 6% were clinically relevant [11]. Ramirez et al. described referral criteria such as negative radiographs, constant pain, neurological abnormalities, and night pain [18]. In line with these criteria, scoliosis observed on plain radiographs was not considered an abnormality. Applying these criteria to our cohort, 15.8% (12 of 76 patients with full available data) showed relevant MRI findings. Several factors may explain the difference. First, Ramirez et al. did not clearly define when an MRI finding was considered abnormal. Second, our retrospective design limited a systematic evaluation of clinical red flags, whereas the prospective design of Ramirez et al. allowed more complete and systematic symptom assessment, minimizing missing data. Third, the variable ‘constant pain’ was excluded from our analyses due to substantial missing data. In contrast, Ramirez et al. reported that constant pain was a highly sensitive predictor of spinal abnormalities, whereas night pain, neurological abnormalities, and scoliosis were less sensitive predictors. This may have limited the validity of the clinical red flags.

In the added-value MRI group, the most common abnormalities were spondylolysis/spondylolisthesis and discopathy, consistent with findings reported in previous studies [10]. However, disc abnormalities are also frequently observed in asymptomatic children [20], likely due to MRI’s high sensitivity [18,21]. These findings suggest that disc abnormalities may not necessarily be clinically relevant, emphasizing the need to combine the medical history and physical examination with MRI findings to avoid misdiagnosis or unnecessary treatment [20].

Most abnormalities were located in the lumbar spine, followed by the thoracic and cervical spines, which is consistent with the distribution reported in an earlier study [22]. Lumbar pain has been identified as a red flag for underlying abnormalities [18,19,23], requiring careful evaluation. Although scoliosis was a common indication for MRI referral, only two cases revealed a secondary cause related to the scoliosis. These findings support the conclusion of Johnson et al., who reported that MRI has limited diagnostic value in patients with idiopathic scoliosis without neurological symptoms or atypical features [24], highlighting the potential overuse of MRI in such cases.

When patients in the added-value MRI group are compared with those in the no-added-value MRI group, neurological abnormalities were identified as one of the statistically significant factors for abnormal MRI findings, consistent with previous studies identifying them as the strongest predictor of clinically relevant MRI findings [23,25]. These results highlight the importance of performing a comprehensive neurological assessment, and in some cases obtaining immediate imaging, when neurological abnormalities are present [25]. The second statistically significant factor was symptom duration. Patients in the added-value MRI group reported shorter duration of pain, whereas chronic back pain (>1 year) was more common in the no-added-value MRI group. Similarly, Ramirez et al. found that symptom duration of less than 3 months was significantly associated with the presence of a specific MRI diagnosis, such as herniated discs, degenerative disc disease, or spondylolysis [18]. In contrast, another study reported that a longer duration of symptoms was associated with MRI abnormalities that led to changes in clinical management [23].

Our stratified analysis demonstrated that MRI diagnostic yield differed significantly by clinical indication. Only 4.4% of MRIs provided added diagnostic value in patients with chronic back pain, compared to 22.8% in patients with other specific indications. These findings support a selective use of MRI in pediatric practice, reserving imaging for cases with specific clinical indications. While only MRI findings that confirmed or established a new diagnosis were classified as providing added diagnostic value in our study, it should be emphasized that MRI-negative cases also have added diagnostic value by excluding serious pathology and offering reassurance in selected clinical scenarios.

No significant associations were found between age or gender and MRI abnormalities. The median age in the added value of MRI group was 13 years. This is in line with the literature, which shows an increasing prevalence of back pain with age [26,27]. Back pain is uncommon in children under 10–12 years but rises in adolescence to near adult rates [3]. Girls reported back pain more frequently than boys, likely due to earlier puberty, hormonal influences, and differences in symptom reporting [3,22,26,28,29,30,31]. However, Dissing et al. did not find a significant association between gender and back pain [32].

Psychological and psychosocial factors could not be included in the analysis due to limited data availability, although they are important risk factors for the development of back pain [28,29,33]. Two of these studies indicated that children whose parents suffer from back pain are more likely to present with back pain themselves [28,33]. Moreover, recent research has identified psychological distress as an independent risk factor for the development of back pain during adolescence [34]. As back pain was frequently observed in our cohort, careful assessment of these factors should be included in the clinical evaluation of children presenting with back pain.

While MRI is used as a valuable diagnostic tool, MRI examination can be a burden for young patients, causing anxiety, distress, and claustrophobia [35]. Younger children often require sedation to prevent movement and ensure high-quality imaging, which is associated with side effects and contributes to increased healthcare costs. Previous studies have reported an increase in the use of sedation and anesthesia for pediatric MRIs, contributing to rising healthcare costs [12,36].

While this study offers valuable insights by addressing spinal MRI abnormalities in children and adolescents across various clinical indications, it has several limitations. First, the retrospective design has led to incomplete and missing data, particularly for key clinical variables, which could limit the robustness of statistical comparisons and lead to gaps or inaccuracies in the analysis. Future research should adopt prospective methodologies to ensure that all factors are thoroughly assessed and documented to minimize data gaps. In addition, the long recruitment period may have introduced technical bias due to advancements in MRI equipment, potentially affecting image quality and interpretation. Finally, larger study populations are recommended in future research to improve reliability.

## 5. Conclusions

This study highlights the low prevalence of clinically relevant spinal abnormalities detected by MRI in children and adolescents. Although most MRIs revealed findings, only a small proportion showed added value from MRI. These findings indicate that a substantial number of MRIs did not reveal new diagnostic or therapeutic information, while placing a physical and emotional burden on pediatric patients and increasing healthcare costs. Patients with clear clinical indications should be prioritized for MRI. In addition, patient characteristics, particularly neurological abnormalities and short symptom duration, should be considered to optimize MRI use. Future research should develop clear criteria for spinal MRI use in children and adolescents to minimize unnecessary imaging, limit patient burden, and optimize healthcare resources.

## Figures and Tables

**Table 1:** Descriptive statistics for all variables (n = 229).

Variables	No. (%/Range)
Age (y)—median	13 (0–16)
Sex	
Girl	151 (65.9)
Boy	78 (34.1)
Night pain *	
No	84 (42.2)
Yes	61 (30.7)
NR	54 (27.1)
Exercise-dependent pain *	
No	40 (20.1)
Yes	130 (65.3)
NR	29 (14.6)
Sharp localization of pain *	
No	165 (82.9)
Yes	26 (13.1)
NR	8 (4.0)
History of trauma	
No	128 (55.9)
Yes	42 (18.3)
NR	59 (25.8)
Neurological abnormality	
No	130 (56.8)
Yes **	74 (32.3)
Radiating pain	56 (75.7)
Upper leg (to the knee)	22 (39.3)
Pelvis	19 (33.9)
Lower leg (below the knee)	18 (32.1)
Arm/hand	3 (5.4)
Shoulder	2 (3.6)
NR	8 (14.3)
Sensory disturbances	24 (32.4)
Pain with moments of pressure	9 (12.2)
Positive Lasègue’s sign	8 (10.8)
Muscle weakness	7 (9.5)
Gait abnormalities	5 (6.8)
Abnormal reflexes	1 (1.4)
NR	25 (10.9)
Duration of pain *	
0–2 weeks	9 (4.5)
2–4 weeks	2 (1.0)
1–3 months	18 (9.0)
3–6 months	19 (9.5)
6–12 months	35 (17.6)
>1 year	109 (54.8)
NR	7 (3.5)

* These variables apply only to patients experiencing back pain (n = 199). ** Multiple responses per patient were possible for the type of neurological abnormality, resulting in a total percentage exceeding 100%. Abbreviations: NR: not reported.

**Table 2:** Indications for MRI request (n = 229).

Indications *	No. (%)
Chronic back pain	73 (31.9)
Neurological abnormalities	42 (18.3)
Scoliosis	41 (17.9)
Congenital vertebral abnormalities	34 (14.8)
Discopathy	33 (14.3)
Malignancy/night pain/bone pathology	28 (12.2)
Post-traumatic back pain	19 (8.3)
Infection	17 (7.4)
Spondylolysis/spondylolisthesis	16 (7.0)
Herniated disc	12 (5.2)
Localized pain	6 (2.6)
Scheuermann’s disease/hyperkyphosis	5 (2.2)
Progressive pain	5 (2.2)
Apophyseal abnormalities/osteochondrosis	3 (1.3)
Other	1 (0.4)

* Multiple responses per patient were possible for the indication for MRI request, resulting in a total percentage exceeding 100%.

**Table 3:** Frequency of MRI findings (n = 229).

Findings *	No.
(1) No finding	89
(2) Spinal incidental finding	64
(3) Confirmed diagnosis with no additional information	71
(4) Confirmed diagnosis/severity with additional information	26
(5) New diagnosis established	21

* Groups 1–5 correspond to categories of MRI findings; multiple responses per patient were possible.

**Table 4:** Radiologist-reported MRI abnormalities (n = 44 patients).

MRI Abnormalities *	No. (%)	C **	T **	L **
Spondylolysis/spondylolisthesis	12 (27.3)	–	–	12
Discopathy	7 (15.9)	–	–	7
Discus bulging/protrusion	6	–	–	6
Intraosseous disc	1	–	–	1
herniation				
Herniated disc	6 (13.6)	–	–	6
Congenital abnormality ***	5 (11.4)	2	2	5
Vertebral anomaly	3	–	–	3
Syringomyelia	2	1	2	–
Normal anatomical	2	–	–	2
variant		–	–	–
Spinal pseudarthrosis	1	1	–	–
Radiculopathy unrelated to a herniated disc	5 (11.4)	–	–	5
Tumour	5 (11.4)	1	1	3
Spondylodiscitis	3 (6.8)	–	–	3
Osteomyelitis	3 (6.8)	–	2	1
Trauma	2 (4.5)	–	1	2
Scoliosis	2 (4.5)	–	2	2
Apophyseal abnormality/osteochondrosis	1 (2.3)	–	–	1
Scheuermann’s disease/hyperkyphosis	1 (2.3)	–	1	1
Non-spinal incidental finding ***	1 (2.3)			
Other abnormality ***	1 (2.3)			
Total radiologist-reported findings	54	3	9	48

* Multiple responses per patient were possible for the type of MRI abnormalities, resulting in a total percentage exceeding 100%. ** Multiple anatomical levels were possible for each abnormality. *** Anatomical levels were not determined for non-spinal incidental findings and other abnormalities. Abbreviations: C: cervical spine; T: thoracic spine; L: lumbar spine.

**Table 5:** Added diagnostic value of spinal MRI by indication group.

Indication Group	No. (%)	Added Value MRIs *	NNI	*p*-Value ^a^
Chronic back pain	45 (19.7)	2 (4.4)	23	0.005
Other specific indications	184 (80.3)	42 (22.8)	4	0.005

^a^ Chi-squared test. * Percentages represent the proportion of MRIs with added diagnostic value within each indication group. Abbreviations: NNI: Number needed to image.

**Table 6:** Comparison of variables between the no added value of MRI group (n = 185) and the added value of MRI group (n = 44).

	No Added Value of MRI (N = 185)			Added Value of MRI (N = 44)			*p*-Value ^a^
**Variable**	No	Yes	NR	No	Yes	NR	
Age (y)—median (range)	14 (0–16)			13 (0–16)			0.185 ^b^
Sex							0.156
Girl Boy	126 (68.1)			25 (56.8)			
	59 (31.9)			19 (43.2)			
Night pain *	74 (45.7)	47 (29.0)	41 (25.3)	10 (27.0)	14 (37.8)	13 (35.1)	0.115
Exercise-dependent pain *	34 (21.0)	105 (64.8)	23 (14.2)	6 (16.2)	25 (67.6)	6 (16.2)	0.794
Sharp localization of pain *	134 (82.7)	23 (14.2)	5 (3.1)	31 (83.8)	3 (8.1)	3 (8.1)	0.216
History of trauma	104 (56.2)	33 (17.8)	48 (25.9)	24 (54.5)	9 (20.5)	11 (25.0)	0.922
Neurological abnormality **	113 (61.1)	51 (27.6)	21 (11.4)	17 (38.6)	23 (52.3)	4 (9.1)	**0.009**
Radiating pain	146 (78.9)	39 (21.1)	–	27 (61.4)	17 (38.6)	–	**0.015**
Sensory disturbances	167 (90.3)	18 (9.7)	–	38 (86.4)	6 (13.6)	–	0.421
Pain with moments of pressure	179 (96.8)	6 (3.2)	–	41 (93.2)	3 (6.8)	–	0.380
Positive Lasègue’s sign	183 (98.9)	2 (1.1)	–	38 (86.4)	6 (13.6)	–	**0.001**
Muscle weakness	181 (97.8)	4 (2.2)	–	41 (93.2)	3 (6.8)	–	0.132
Gait abnormalities	183 (98.9)	2 (1.1)	–	41 (93.2)	3 (6.8)	–	**0.050**
Abnormal reflexes	184 (99.5)	1 (0.5)	–	44 (100)	0	–	1.000
Duration of pain *							**0.002 ^b^**
0–2 weeks	4 (2.5)			5 (13.5)			
2–4 weeks	0			2 (5.4)			
1–3 months	14 (8.6)			4 (10.8)			
3–6 months	15 (9.3)			4 (10.8)			
6–12 months	28 (17.3)			7 (18.9)			
>1 year	94 (58.0)			15 (40.5)			
NR	7 (4.3)			0			

^a^ Chi-squared test or Fisher/Freeman–Halton exact test if expected counts < 5. ^b^ Mann–Whitney U-test. * These variables apply only to patients experiencing back pain (n = 199). ** Multiple responses per patient were possible for the type of neurological abnormality, resulting in a total percentage exceeding 100%. Age, sex, and pain duration are reported for each group. For all other variables, patient counts are provided per response category (No/Yes/NR). Values are presented as numbers and percentages unless otherwise indicated.

## Data Availability

The data presented in this study are available on request from the corresponding author.

## Supplementary Materials

The following supporting information can be downloaded at https://www.mdpi.com/article/10.3390/children13020294/s1. Table S1: Radiologist-reported MRI abnormalities (n = 140 patients); Table S2: Overview of clinical management and follow-up policy (n = 229).
