# Supplementary material for: Abnormalities on Spinal Magnetic Resonance Imaging in Children and Adolescents: A Two-Center Retrospective Cohort Study
Source: Children (Basel). 2026 Feb 20;13(2):294. doi: 10.3390/children13020294 (PMC12939223; doi:10.3390/children13020294)
Supplement: Supplementary file 1 [file children-13-00294-s001.zip › children-4135859-supplementary.pdf]

Supplementary Materials

Table S1. Radiologist-reported MRI abnormalities (n = 140 patients).

MRI abnormalities*	No. (%)	C**	T**	L**
Scoliosis	50 (35.7)	–	47	41
Discopathy	47 (33.6)	2	9	47
Discus degeneration	30 (63.8)	1	7	23
Discus bulging/ protrusion	22 (46.8)	1	2	23
Other	1 (2.1)	–	–	1
Congenital abnormality***	28 (20.0)	4	11	19
Normal anatomical variant	12 (42.9)	–	3	9
Vertebral anomaly	9 (32.1)	2	1	6
Other	6 (21.4)	1	3	3
Syringomyelia	5 (17.9)	1	4	1
Schmorl's nodes	17 (12.1)	–	8	12
Spondylolysis/ spondylolisthesis	13 (9.3)	–	–	13
Herniated disc	8 (5.7)	–	–	8
Non-spinal incidental finding***	7 (5.0)			
Apophyseal abnormality/ osteochondrosis	6 (4.3)	–	3	4
Radiculopathy unrelated to herniated disc	5 (3.6)	–	–	5
Tumour	5 (3.6)	1	1	3
Trauma	4 (2.9)	–	3	2
Scheuermann's disease/ hyperkyphosis	4 (2.9)	–	3	2
Spondylodiscitis	3 (2.1)	–	–	3
Osteomyelitis	3 (2.1)	–	2	1
Limbus vertebrae	2 (1.4)	–	–	2
Other abnormalities***	1 (0.7)			
Total radiologist-reported findings	203	7	87	162

* Multiple responses per patient were possible for the type of MRI abnormalities, resulting in a total percentage exceeding 100%. ** Multiple anatomical levels were possible for each abnormality. *** Anatomical levels were not determined for non-spinal incidental findings and other abnormalities. Abbreviations: MRI: Magnetic Resonance Imaging; C: cervical spine; T: thoracic spine; L: lumbar spine.

Table S2. Overview of clinical management and follow-up policy (n = 229).

**Policy***	**No. (%)**
Referral*	184 (80.3)
Physical therapist	91 (49.5)
Mensendieck and/or Cesar therapist	27 (14.7)
Rehabilitation physician	21 (11.4)
Neurologist	17 (9.2)
Psychosomatic physical therapist	16 (8.7)
Orthopedic surgeon in academic hospital	15 (8.2)
Other	14 (7.6)
Pediatrician	12 (6.5)
Scoliosis physical therapist	10 (5.4)
Sports medicine physician	5 (2.7)
Manual therapist	2 (1.1)
Back school	1 (0.5)
Pain clinic	1 (0.5)
Psychologist	1 (0.5)
Follow-up consultation	60 (26.2)
Treatment*	46 (20.1)
Brace or corset	32 (69.6)
Medication	14 (30.4)
Surgical intervention	2 (4.3)
Other	1 (2.1)
Further examination*	41 (17.9)
Laboratory testing	23 (56.1)
Bone scintigraphy with dynamic injection and SPECT	11 (26.8)
CT scan	11 (26.8)
Bone scintigraphy with dynamic injection	10 (24.4)
Other	2 (4.9)
Echo	1 (2.4)
End of treatment	24 (10.5)
NR	2 (0.9)
Other	1 (0.4)

* Multiple responses per patient were possible for policy, referral, treatment and further examination, resulting in a total percentage exceeding 100%. Abbreviations: SPECT: Single Photon Emission Computed Tomography; CT: Computed Tomography; NR: not reported.
